# Autochthonous outbreak of respiratory diphtheria caused by *Corynebacterium diphtheriae*, Germany, September 2024

**DOI:** 10.2807/1560-7917.ES.2025.30.27.2500116

**Published:** 2025-07-10

**Authors:** Anja Berger, Alexandra Dangel, Katja Bengs, Sandra Schlotmann, Peter Thomaßen, Claudia Maday, Carmen Rubach, Inas Abdelgawad, Gabriele Namaschk, Lena Schneider, Delphine Perriat, Franziska Badenschier, Cornelius Rau, Mark Muscat, Andreas Sing

**Affiliations:** 1 National Consiliary Laboratory for Diphtheria, Oberschleißheim, Germany; 2 European Union (EU) Reference Laboratory Diphtheria & Pertussis (EURL-PH-DIPE), Oberschleißheim, Germany; 3 World Health Organization (WHO) Collaborating Centre for Diphtheria, Oberschleißheim, Germany; 4 Department of Public Health Microbiology, Bavarian Health and Food Safety Authority, Oberschleißheim, Germany; 5 NGS Core Unit, Bavarian Health and Food Safety Authority, Oberschleißheim, Germany; 6 Public Health Authority Havelland, Rathenow, Brandenburg, Germany; 7 Forensic Medicine and Clinical Toxicology Department, Faculty of Medicine, Cairo University, Al Kasr Al Aini, Cairo, Cairo Governorate, Egypt; 8 Public Health Authority Berlin-Spandau, Berlin, Germany; 9 Department of Infectious Disease Epidemiology, Postgraduate Training for Applied Epidemiology, Robert Koch Institute (RKI), Berlin, Germany; 10 ECDC Fellowship Programme, EPIET Associated Programme, European Centre for Disease Prevention and Control (ECDC), Stockholm, Sweden; 11 Department of Infectious Disease Epidemiology, Robert Koch Institute (RKI), Berlin, Germany; 12 Vaccine-Preventable Diseases and Immunization Programme, World Health Organization Regional Office for Europe, Copenhagen, Denmark

**Keywords:** diphtheria, toxigenic, Corynebacterium diphtheriae, WGS, NGS, MLST, outbreak, typing, migrants, Ill-Housed Persons

## Abstract

In September 2024, a school-aged child (P1), unvaccinated against diphtheria, was hospitalised with severe respiratory diphtheria caused by toxigenic *Corynebacterium diphtheriae.* P1 subsequently died from the disease. The child’s mother (P2) had pharyngitis 9 days before the onset of symptoms of P1 and subsequently tested positive for *C. diphtheriae*. In multilocus sequence typing (MLST), the *C. diphtheriae* isolates from P1 and P2 were of sequence type (ST) 574. In core genome (cg)MLST, they were clonal, suggesting recent human-to-human transmission. This indicates the first autochthonous respiratory diphtheria outbreak by toxigenic *C. diphtheriae* in Germany since 1984 with epidemiologically- and molecularly-confirmed transmission. Furthermore, the isolates were close to isolates from patients with cutaneous diphtheria among people experiencing homelessness in two major German cities in 2023 and 2024, and to isolates from an outbreak among newly arriving migrants across several European countries, including Germany, detected in 2022. This indicates transmission beyond vulnerable groups. Our findings illustrate the potential of *C. diphtheriae* spreading further from patients with cutaneous diphtheria and even causing outbreaks of respiratory diphtheria. Given the potentially serious complications of respiratory diphtheria, including death, equitably achieving and maintaining high vaccination coverage among the whole population, especially among vulnerable people is essential.

**Key public health message**

**What did you want to address in this study and why?**

Respiratory diphtheria is a severe vaccine-preventable disease, caused by diphtheria-toxin producing (i.e. toxigenic) corynebacteria*.* We report the first outbreak of respiratory diphtheria caused by *Corynebacterium diphtheriae* where transmission occurred within Germany, in 40 years. One unvaccinated child died in this outbreak. We investigated this outbreak to understand how the pathogen spread, who was affected and how to prevent more people from getting infected.

**What have we learnt from this study?**

The outbreak investigation shows that the diphtheria bacterium spread from newly arriving migrants to residents in Germany: first to people experiencing homelessness in two metropolitan areas in Germany, and then to a local family. Results also show that a person with diphtheria of the skin may infect another person who then might develop respiratory diphtheria which is more serious and can lead to death.

**What are the implications of your findings for public health?**

Whole genome sequencing can support the detection and investigation of outbreaks. People who are vulnerable because of barriers to accessing healthcare services need better access to vaccinations, diagnostics and treatment. This is especially important for newly-arriving migrants and people experiencing homelessness. The more people are vaccinated against diphtheria the easier it is to stop outbreaks and to prevent future ones.

## Background

Diphtheria is a vaccine-preventable disease with cutaneous or respiratory manifestation, caused by diphtheria toxin-producing (toxigenic) corynebacteria, mainly *Corynebacterium diphtheriae* or zoonotic *Corynebacterium ulcerans*. The pathogen is usually transmitted through contact with a patient or a person carrying the bacterium in their throat or on their skin. Classical respiratory diphtheria results from local and systemic spread of diphtheria toxin causing a pseudomembranous inflammation of the upper respiratory tract as well as acute systemic toxicity, myocarditis and polyneuritis. The incubation period is usually 1–5 days with a maximum of 10 days. Depending on the settings, the complications can give rise to a case fatality rate of 5–30% [1], even with treatment. The period of communicability lasts until virulent corynebacteriae have disappeared from the lesions they produce, usually in ≤ 2 weeks, and seldom > 4 weeks for respiratory diphtheria in untreated people [1,2].

In countries with adequate vaccination coverage, diphtheria is rare. In Germany, the Standing Committee on Vaccination (STIKO) recommends basic immunisation against diphtheria at the age of 2, 4 and 11 months, followed by booster vaccinations at the age of 5–6 years and 9–16 years [3]. In adulthood, a booster vaccination is recommended every 10 years. Vaccination coverage for the primary vaccination series against diphtheria at the age of 6 years is high (89%) [4].

Diphtheria outbreaks occur rarely in Germany and sporadic diphtheria cases are uncommon with only a few annually reported cases. According to the German Infection Protection Act, mandatory notification of all toxigenic *C. diphtheriae* is required in Germany since 2001 and was extended to all toxigenic *Corynebacterium* spp. in 2017. Upon clinical suspicion or diagnosis, the treating physician, and upon laboratory detection, laboratories diagnosing toxigenic corynebacteria are obliged to notify the responsible local public health authority. Recommendations include submission of isolates from suspected cases of diphtheria to the German National Consiliary Laboratory for Diphtheria (GNCLD) in Oberschleißheim, for further confirmatory testing including testing for toxigenicity and molecular typing, e.g. whole genome sequencing (WGS) [5].

## Outbreak detection

In September 2024, a school-aged child (P1) presented with clinical signs of tonsillitis to a general practitioner. On the next day, and with clinical deterioration, P1 was admitted to a primary hospital, now presenting with severe tonsillitis, a pseudomembrane and lymph node swelling. P1 was unvaccinated against diphtheria. A nasopharyngeal swab obtained on hospital admission grew *C. diphtheriae*. The isolate was sent to the GNCLD for toxigenicity testing, where it was named KL3499 and tested toxigenic. Based on the clinical suspicion of diphtheria, the patient received antibiotic therapy with penicillin and diphtheria antitoxin (DAT) under intensive care observation and was transferred to a tertiary referral hospital due to severe deterioration of health including cardiac, renal and respiratory involvement requiring invasive ventilation and intensive care. P1 eventually succumbed to the illness in January 2025.

The event was considered unusual as it involved the death of a child in the local population. For comparison: three respiratory diphtheria cases caused by *C. diphtheriae* were notified in Germany between 2001 and 2022 – all of them adults, two infections were imported, none fatal. In 2022, there were 13 cases of respiratory diphtheria as part of an international outbreak of diphtheria among newly-arriving migrants – mainly young adults, all infections were imported, no deaths occurred in Germany.

Here, we describe the epidemiological, microbiological and molecular outbreak investigations of the fatal case of respiratory diphtheria caused by toxigenic *C. diphtheriae* of P1 and their mother. Whole genome sequencing results show similarity of the outbreak isolates with isolates from people experiencing homelessness in Germany in 2023 and 2024 as well as from newly arriving migrants linked to the international outbreak in 2022.

## Methods

### Outbreak case definition

As a confirmed case, we defined any person with laboratory-confirmed toxigenic *C. diphtheriae* and an epidemiological link to the index case (P1).

### Microbiological and molecular investigations

Toxigenicity testing at the GNCLD was done by real-time PCR [6], lateral flow immunoassay (LFIA) [7] and an optimised Elek test [8] using methods as described previously.

Further microbiological analyses included biochemical differentiation (API Coryne, Biomérieux, Marcy-l'Étoile, France) and matrix-assisted laser desorption/ionization time-of-flight mass spectrometry (MALDI-TOF MS, MALDI Biotyper; Bruker Daltonics, Billerica, the United States (US)) using methods as described previously [9]. Antimicrobial drug susceptibility testing of the isolate was performed on Mueller-Hinton agar supplemented with 5% lysed horse blood and 20 mg/L beta-nicotinamide adenine dinucleotide (NAD) (MH-F agar) by agar diffusion test and read after 18 ± 2 hours incubation at 35 ± 1°C in a 5% CO_2_ atmosphere. Disc diffusion diameters were determined according to the European Committee on Antimicrobial Susceptibility Testing (EUCAST) guidelines for *C. diphtheriae* [10].

Whole genome sequencing was carried out after DNA extraction with Beckman Coulter Genfind V3 kit (Beckman Coulter, Brea, US), using library preparation with the Illumina DNA prep kit and Illumina paired-end next generation sequencing (NGS) (Illumina, San Diego,US). Multilocus sequence typing (MLST) based on seven housekeeping loci [11] and core genome (cg) MLST analysis based on a scheme of 1,553 *C. diphtheriae*-specific target loci [12] were carried out with the NGS data using Ridom SeqSpehre + software (Ridom GmbH, Munster, Germany).

### Epidemiological investigations

The responsible local public health authorities initiated public health measures including contact tracing and throat swab sampling from household members and other close contacts following German national recommendations [5]. Further, epidemiological investigations included the matching of laboratory isolates with cases notified under the German Infection Protection Act to consolidate case information from epidemiological investigations.

## Results

Outbreak investigations resulted in the detection of one additional case with an epidemiological link to P1, i.e. the child’s mother (P2). Furthermore, molecular analyses suggest possible links to other diphtheria cases among vulnerable groups, namely people experiencing homelessness and newly arriving migrants in Germany.

### Microbiological results

The isolates KL3499 (P1) and KL3502 (P2) were both tested toxigenic by the three methods mentioned above [6-8] and identified as *C. diphtheriae* biovar *mitis* by biochemical differentiation (API Coryne code 1010324) and MALDI-TOF analysis [9]. Both isolates were sensitive to penicillin G (susceptible, increased exposure), meropenem, erythromycin, clindamycin, tetracycline, rifampicin, linezolid and ciprofloxacin (susceptible, increased exposure), but resistant against cotrimoxazole [10].

### Epidemiological and molecular findings

Epidemiological investigations found that P1 had no history of travel abroad and no contact to known diphtheria cases. P2 worked in child care, fell ill with pharyngitis 9 days before P1 became ill, and was on sick leave for 5 days. At that point, a throat swab arranged by a general practitioner was only examined for streptococci and tested negative. The pharyngeal swab obtained during contact tracing by the local public health authority, 2 days after hospital admission of P1, was positive for *C. diphtheriae*. The isolate (KL3502) was identified as toxigenic and exhibited the same antimicrobial resistance profile as the isolate of P1 after being sent to the GNCLD for further analysis and characterisation. P2 had received the complete set of vaccine doses according to the latest German vaccination recommendations [3], including a three-dose primary series of diphtheria-containing vaccine followed by booster doses, although the most recent booster vaccination was in 2010, i.e. not up to date anymore. P2 had no history of travelling abroad, no contacts to known diphtheria cases, people experiencing homelessness or migrants newly arriving in Germany before her pharyngitis.

For further outbreak investigation, a NGS data analysis was performed comparing both *C. diphtheriae* isolates (KL3499 and KL3502) of P1 and P2, respectively. Classical MLST based on seven housekeeping loci [11], extracted from the WGS data yielded sequence type (ST) 574. This ST was first detected in Germany in 2022 among migrants arriving in Germany [12]. It had been designated in the pubMLST database (https://bigsdb.pasteur.fr/diphtheria/) in December 2021 in a sample from India. Core genome MLST [12] revealed only one allele distance (AD) between the two isolates, thereby confirming clonality (Figure).

**Figure:**
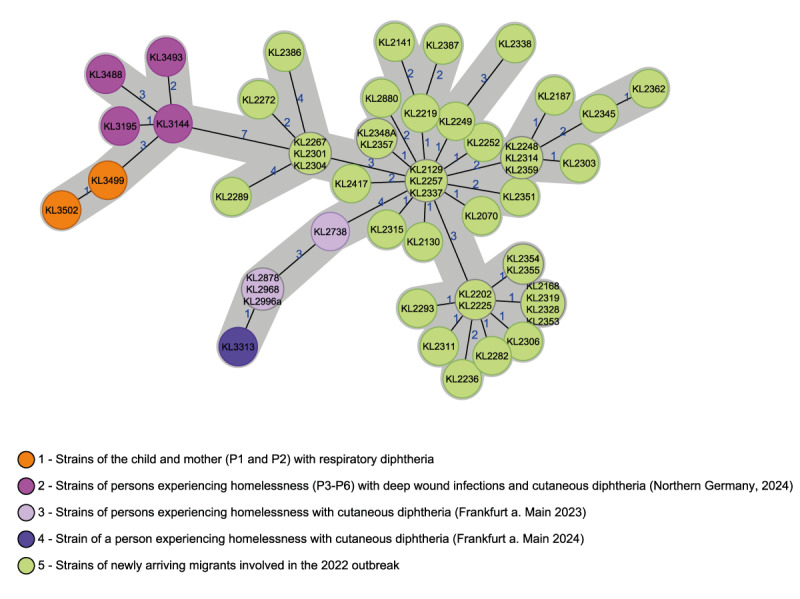
Minimum spanning tree of core genome multilocus sequence typing analysis of isolates of *Corynebacterium diphtheriae,* Germany, 2022–2024 (n = 54)

cg MLST: core genome multilocus sequence typing.

cg MLST analysis was based on a scheme of 1,553 *Corynebacterium diphtheriae*-specific target loci. Isolates from the two outbreak cases and genetically related cases of a cluster from the same sequence type, comprising mainly isolates obtained from newly arriving migrants, including two subclusters of isolates from persons experiencing homelessness.

Both isolates showed close genetic relationship (10–16 AD) to a cluster of the same ST 574 from the 2022 European diphtheria outbreak among migrants [12-14]. Noteworthy, the isolate from P1 showed very few AD to isolates from other recent diphtheria cases in Germany. It had an AD of only 3–4 to four recent isolates (KL3144, KL3195, KL3488, KL3493) of cutaneous diphtheria among people experiencing homelessness (P3-P6) from the same metropolitan area where P1 and P2 attended school and work (Table). In addition, it had an AD of 15–17 to an additional sub-cluster of persons experiencing homelessness from the metropolitan area of Frankfurt am Main in the federal state of Hesse [15] (Figure). In the Supplemental Table, cgMLST-derived AD of all analysed isolates to P1 are presented. P2 showed one more allele distance than P1 to the other isolates.

**Table:** Characteristics of patients with toxigenic *Corynebacterium diphtheriae* isolates, a metropolitan area in Germany, 2024 (n = 6)

Patient	Isolate ID	Month of sampling	AMR	ST	CT	Patient age group (years)	Country of origin	Clinical manifestation	Vaccination against diphtheria^a^	Hospitalisation	Exposures of interest
P1	KL3499	Sep	Cotrimoxazole	574	79	10-14	Germany	Severe respiratory diphtheria	Unvaccinated	Yes	Visited city where cases of diphtheria among people experiencing homelessness had occurred in 2024
P2	KL3502	Sep				50-59	Germany	Mild respiratory diphtheria	Basic vaccination completed, subsequent boosters, last booster vaccination in 2010	No	Mother of P1, no other relevant exposure reported
P3	KL3144	Jul				50-59	Unknown	Cutaneous diphtheria	Unknown	Yes	Experiencing homelessness
P4	KL3195	Feb				60-69				Yes, reason unknown	
P5	KL3488	Sep				40-49				Yes	
P6	KL3493	Sep				40-49				Yes, for another reason	

AMR: antimicrobial resistance; CT: complex type; GNCLD: German National Consiliary Laboratory on Diphtheria; ID: identification code; ST: sequence type; R: resistant.

^a^ Vaccination status including any vaccine containing diphtheria toxoid.

Patients P3-P6 had deep wound infections. Isolates of *C. diphtheriae* from the wounds were sent to the GNCLD between January and September 2024. All isolates were toxigenic and cotrimoxazole-resistant, as the isolates KL3499 and KL3502 from P1 and P2 (Table).

## Outbreak control measures

Public health measures around the outbreak focused on contact tracing. Nine household members received post-exposure prophylaxis (PEP) consisting of azithromycin or erythromycin. The local public health authority responsible for the school of P1 and workplace of P2 arranged swab taking and recommended administration of PEP for all close contacts of P1 at school and of P2 at work. All close contacts of P1 and P2 and their household members tested negative for *C. diphtheriae*. No secondary case was detected.

## Discussion

Diphtheria outbreaks occur rarely in Germany. The last autochthonous respiratory diphtheria outbreak was reported in the town of Wuppertal between 1982 and 1984 [16]. In 2018, an outbreak comprising two siblings with imported cutaneous diphtheria caused by *C. diphtheriae* was reported [17]. In summer 2022, German public health authorities observed an increase of notified diphtheria cases caused by *C. diphtheriae* associated with the European-wide outbreak among people seeking asylum [12-14]. In line with the fact that *C. diphtheriae* cases are often travel-associated, both outbreaks exclusively described patients with imported diphtheria and no secondary cases were identified in Germany but the second sibling [17]. In October 2023, an outbreak investigation among people experiencing homelessness in a German city documented community transmission of cutaneous diphtheria caused by toxigenic *C. diphtheriae* [15] for the first time, in contrast to previously seen cases of non-toxigenic *C. diphtheriae* in a similar population [18].

The new outbreak described here presents the first established autochthonous outbreak of respiratory diphtheria caused by toxigenic *C. diphtheriae,* possibly linked to transmission among people experiencing homelessness in other German cities or migrants with imported diphtheria. The results of the outbreak investigation have several implications and consequences for public health measures.

Sequencing (WGS) confirmed the autochthonous diphtheria outbreak with 1 AD between the isolates of P1 and P2 and allowed the identification of just a few AD from the isolates of P1, P2 and P3–P6 among people experiencing homelessness, thus suggesting a possible link. The isolates of P1–P6 were typed as ST 574, an ST not detected in Germany before 2022. However, as during the pan-European outbreak, a cluster developed within this ST. Thereby, ST 574 isolates make up 27% of the European entries within the pubMLST database (292 out of 1,077 as of 23 April 2025). However, as uploading to the pubMLST database is not mandatory, and outbreak isolates are more likely to be uploaded due to public health interest than sporadic isolates, this percentage may be biased.

During the outbreak investigation of the 2022–2023 diphtheria cases imported to Germany, no secondary cases among the wider population could be identified. However, WGS results highlight the possible link of imported diphtheria cases with community transmission among people experiencing homelessness and the described respiratory diphtheria outbreak [15]. Although there are no hard thresholds for the number of AD with which isolates are assigned to a cluster or not, previous studies have shown that allelic and SNP distances are usually in a similar range [18]. A relationship can be assumed for distances, such as those between the isolates of the main node of the cluster and especially within the subcluster of isolates of the same region, [12,14,15,19,20]. For the specific cgMLST scheme used in this study [18], we applied an empirical cluster threshold of 14. Taking these facts and an estimated substitution size of 1.67 × 10^−6^ per site and year into account [21], the small (3–4) to medium (10–16) AD in the minimum spanning tree (MST) indicate of persistent transmission over the timespan of approximately 2 years, considering an expansion from the main node of the MST. In the absence of clear epidemiological links, however, direct correlations could not be established. However, the phylogeny cannot give the complete picture, and isolates, particularly from vulnerable groups such as people experiencing homelessness and from vaccinated persons with asymptomatic or sub-clinical (mild) respiratory diphtheria, might be missing. The analysis shows, however, that *C. diphtheriae* transmission occurs in different geographic areas and poses a risk to different groups vulnerable because of barriers to accessing healthcare, such as migrants, people experiencing homelessness and all unvaccinated individuals.

Notably, a similar finding was reported from Basel, Switzerland: two genetically identical strains of ST 574 from two over 70-years-old adults showed only 6–16 AD to a cluster of strains obtained in 2022 from a federal asylum centre in the same area, with no epidemiological links between the two patients or the migrant population [19]. This underlines the importance of ensuring early and timely diagnosis of imported diphtheria to prevent further transmission. For instance, as part of initial medical examinations, newly arriving migrants with skin lesions should be routinely tested for *Corynebacterium* spp. and receive, if necessary, antimicrobial treatment and instructions on infection prevention to avoid further transmission [13,22].

Furthermore, it is essential to consider that the number of unreported cases is not known and that both groups – newly arriving migrants and people experiencing homelessness – are not mutually exclusive. For a variety of reasons, migrants might experience homelessness. Thus, additional active case finding among people experiencing homelessness in the cities linked to the outbreak of mainly cutaneous diphtheria among newly arriving migrants across several European countries [12-14] could ensure detection of undiagnosed diphtheria cases or pathogen carriage and adequate treatment. At the same time, offering vaccination checks and services contributes to the protection of individuals as well as the community. Given the vulnerabilities of the aforementioned groups, such as barriers to access healthcare, vaccination services should be part of any medical outreach [23].

The two outbreak cases lived in relative geographic proximity to the location of the genetically closely linked cutaneous diphtheria cases. Nevertheless, despite extensive case finding, this outbreak investigation could not establish an epidemiological link between the respiratory cases and cutaneous cases among people experiencing homelessness. Neither P1 nor P2 reported any contact to people experiencing homelessness or newly-arriving migrants.

Although the isolate from P1 seems to be genetically closer related to the diphtheria cases among people experiencing homelessness than P2, the timeline of symptom onset with the P2’s symptoms appearing 9 days before P1’s may indicate transmission from mother to child. The clinical course suggests that P2 might have had a less severe form of respiratory diphtheria because of her vaccinations and thus unconsciously passed the pathogen to P1. This is also in line with longer unusual but observed incubation periods beyond 5 days. But since the swab from P2 was taken 2 days after hospital admission of P1, it is also possible that P2 was colonised through the contact with P1. However, it cannot be completely ruled out that P1 was infected first and transmitted *C. diphtheriae* to P2 who then displayed respiratory colonisation with *C. diphtheriae*. As discussed above, the phylogeny does not include all possible isolates, as a certain number of cases may neither be reported nor analysed. Hence, it can be assumed that transmission has taken place, but the discussed options are the most likely of several possibilities of how the pathogen may have spread.

P1 and P2 had respiratory diphtheria while the patients with the genetically closest isolates and living in the geographically nearest area to them had cutaneous diphtheria. The common understanding is that cases of cutaneous diphtheria lead to cutaneous diphtheria in other persons via direct or indirect contact transmission. However, the findings here show that it might be possible that cutaneous diphtheria leads to respiratory diphtheria. Double manifestations may play a role as some migrants in the 2022 outbreak had cutaneous diphtheria combined with colonisation of the respiratory tract or mild respiratory symptoms [12]. This stresses the need to ensure adequate protection for people with high risk of exposure to cutaneous diphtheria such as people living or working in shelters or asylum reception centres.

In a world connected by travel, migration and other forms of person movements, pathogens causing vaccine-preventable diseases can be imported at any time. If vaccination coverage levels in the population are inadequately low, this can pose a risk, particularly to un- or undervaccinated individuals. Therefore, providing access to vaccinations, debunking misinformation, addressing causes of low vaccine acceptance and skepticism to ensure high vaccine uptake is of utmost importance to protect individuals and communities. The best protection against diphtheria is the diphtheria toxoid vaccine based on the diphtheria toxin of *C. diphtheriae*.

Marginalised groups such as migrants and people experiencing homelessness often have restricted access to health services, resulting in low vaccination coverage. Therefore, it is essential to ensure free and timely access to vaccination services, e.g. by offering vaccination for all migrants or asylum-seekers upon arrival in Germany, as formally guaranteed by the Asylum Seekers Benefits Act, or by conducting targeted booster vaccination campaigns for people experiencing homelessness.

In addition, routine vaccination efforts should be intensified, e.g. during the school entry examinations for children or through health check-ups for adults. Although vaccination levels in Germany are higher for school-aged children (89% at the age of 6 years), timeliness of vaccination is a concern, leaving many young children at risk [4]. Only 64% of German children have received three doses by the age of 15 months, and only 77% are fully immunised against diphtheria by 24 months [4]. In adults, only 53% have received a booster vaccination within the last 10 years [24]. Similarly, anti-diphtheria seroprotection rates have been shown to decrease, especially in older age groups in many European countries [24-27]. Given the increasing life expectancy worldwide the need for booster doses should be evaluated in the long term [28].

## Conclusion

The most important measure for controlling-diphtheria and preventing outbreaks remains high coverage with a diphtheria targeting vaccine in all population groups. Moreover, adequate surveillance systems with laboratory diagnostic capacity are necessary to ensure the detection of people with diphtheria, particularly to identify and respond to autochthonous transmission. Outbreaks, like the one described here, should be a reminder of the importance of vaccines in preventing morbidity and mortality resulting from vaccine-preventable diseases – even those that were long deemed forgotten and disappearing.

## Data Availability

Whole genome sequencing (WGS) data are available under BioProject ID PRJNA1176523 at https://www.ncbi.nlm.nih.gov/bioproject/PRJNA1176523. Detailed accession numbers are given in the Supplemental Table.

## Supplementary Data

126000 Supplement
