# Supplementary material for: Autochthonous outbreak of respiratory diphtheria caused by Corynebacterium diphtheriae, Germany, September 2024
Source: Euro Surveill. 2025 Jul 10;30(27):2500116. doi: 10.2807/1560-7917.ES.2025.30.27.2500116 (PMC12262112; doi:10.2807/1560-7917.ES.2025.30.27.2500116)
Supplement: Supplement [file 25-00116_SING_Supplement.pdf]

SUPPLEMENTAL MATERIAL

This supplementary material is hosted by *Eurosurveillance* as supporting information alongside the article "First outbreak of respiratory diphtheria caused by Corynebacterium diphtheriae in forty years in Germany, September 2024", on behalf of the authors, who remain responsible for the accuracy and appropriateness of the content. The same standards for ethics, copyright, attributions and permissions as for the article apply. Supplements are not edited by *Eurosurveillance* and the journal is not responsible for the maintenance of any links or email addresses provided therein."

SUPPLEMENTAL TABLE: Analysed isolates with allelic distances to the child´s isolate (KL3499) and NCBI accession numbers of genomic sequences. GNCLD: German National Consiliary Laboratory on Diphtheria; AD: Allelic Distance; ST: sequence type; CT: complex type

Sample ID GNCLD (Patient)	AD to KL3499	MLST ST	cgMLST CT	Collection Year	epidemiological group	Bioproject	Biosample Accession
KL3499 (P1)	-	574	79	2024	child with diphtheria	PRJNA1176523	SAMN44405808
KL3502 (P2)	1	574	79	2024	mother of child with diphtheria	PRJNA1176523	SAMN44405809
KL3144 (P3)	3	574	79	2024	person experiencing homelessness Northern Germany	PRJNA1176523	SAMN44451175
KL3493 (P6)	3	574	79	2024	person experiencing homelessness Northern Germany	PRJNA1176523	SAMN44451178
KL3195 (P4)	4	574	79	2024	person experiencing homelessness Northern Germany	PRJNA1176523	SAMN44451176
KL3488 (P5)	4	574	79	2024	person experiencing homelessness Northern Germany	PRJNA1176523	SAMN44451177
KL2304	10	574	79	2022	migrant	PRJNA898270	SAMN36271236
KL2301	10	574	79	2022	migrant	PRJNA898270	SAMN36271233
KL2267	10	574	79	2022	migrant	PRJNA898270	SAMN36271219
KL2272	10	574	79	2022	migrant	PRJNA898270	SAMN36271222
KL2129	11	574	79	2022	migrant	PRJNA898270	SAMN31602464
KL2337	11	574	79	2022	migrant	PRJNA898270	SAMN36271256
KL2257	11	574	79	2022	migrant	PRJNA898270	SAMN36271212
KL2219	12	574	79	2022	migrant	PRJNA898270	SAMN31602493
KL2130	12	574	79	2022	migrant	PRJNA898270	SAMN31602465
KL2315	12	574	79	2022	migrant	PRJNA898270	SAMN36271244
KL2289	12	574	79	2022	migrant	PRJNA898270	SAMN36271227
KL2357	12	574	79	2022	migrant	PRJNA898270	SAMN36271268
KL2249	12	574	79	2022	migrant	PRJNA898270	SAMN36271208
KL2070	12	574	79	2022	migrant	PRJNA898270	SAMN31602461
KL2348A	12	574	79	2022	migrant	PRJNA898270	SAMN36271263
KL2252	12	574	79	2022	migrant	PRJNA898270	SAMN36271209
KL2880	13	574	79	2023	migrant	PRJNA1139060	SAMN42749342
KL2351	13	574	79	2022	migrant	PRJNA898270	SAMN36271264
KL2417	13	574	79	2022	migrant	PRJNA1139060	SAMN42749338

Sample ID GNCLD (Patient)	AD to KL3499	MLST ST	cgMLST CT	Collection Year	epidemiological group	Bioproject	Biosample Accession
KL2359	13	574	79	2022	migrant	PRJNA898270	SAMN36271270
KL2314	13	574	79	2022	migrant	PRJNA898270	SAMN36271243
KL2248	13	574	79	2022	migrant	PRJNA898270	SAMN36271207
KL2303	14	574	79	2022	migrant	PRJNA898270	SAMN36271235
KL2387	14	574	79	2022	migrant	PRJNA898270	SAMN36271275
KL2386	14	574	79	2022	migrant	PRJNA898270	SAMN36271274
KL2141	14	574	79	2022	migrant	PRJNA898270	SAMN31602468
KL2187	14	574	79	2022	migrant	PRJNA898270	SAMN31602481
KL2202	14	574	79	2022	migrant	PRJNA898270	SAMN31602487
KL2225	14	574	79	2022	migrant	PRJNA898270	SAMN31602497
KL2345	15	574	79	2022	migrant	PRJNA898270	SAMN36271261
KL2353	15	574	79	2022	migrant	PRJNA898270	SAMN36271265
KL2328	15	574	79	2022	migrant	PRJNA898270	SAMN36271251
KL2319	15	574	79	2022	migrant	PRJNA898270	SAMN36271245
KL2738	15	574	79	2023	person experiencing homelessness Frankfurt a.M.	PRJNA1139060	SAMN42749339
KL2338	15	574	79	2022	migrant	PRJNA898270	SAMN36271257
KL2306	15	574	79	2022	migrant	PRJNA898270	SAMN36271238
KL2311	15	574	79	2022	migrant	PRJNA898270	SAMN36271241
KL2168	15	574	79	2022	migrant	PRJNA898270	SAMN31602476
KL2354	15	574	79	2022	migrant	PRJNA898270	SAMN36271266
KL2293	15	574	79	2022	migrant	PRJNA898270	SAMN36271230
KL2355	15	574	79	2022	migrant	PRJNA898270	SAMN36271267
KL2282	15	574	79	2022	migrant	PRJNA898270	SAMN36271224
KL3313	16	574	79	2024	person experiencing homelessness Frankfurt a.M.	PRJNA1176523	SAMN44451179
KL2878	16	574	79	2023	person experiencing homelessness Frankfurt a.M.	PRJNA1139060	SAMN42749341
KL2362	16	574	79	2022	migrant	PRJNA898270	SAMN36271272
KL2236	16	574	79	2022	migrant	PRJNA898270	SAMN36271205
KL2968	16	574	79	2023	person experiencing homelessness Frankfurt a.M.	PRJNA1139060	SAMN42749343
KL2996a	17	574	79	2023	person experiencing homelessness Frankfurt a.M.	PRJNA1139060	SAMN42749344
